# Association of predicted body composition with occurrence of atrial fibrillation

**DOI:** 10.3389/fcvm.2023.1159087

**Published:** 2023-10-10

**Authors:** Ho Geol Woo, Min Kyoung Kang, Tae-Jin Song

**Affiliations:** ^1^ Department of Neurology, Kyung Hee University College of Medicine, Kyung Hee University Hospital, Seoul, Republic of Korea; ^2^ Department of Neurology, Seoul Hospital, Ewha Womans University College of Medicine, Seoul, Republic of Korea

**Keywords:** body composition, body mass index, atrial fibrillation, big data, analysis

## Abstract

**Background:**

Body mass index (BMI) is insufficient evidence as a risk factor for numerous health disorders. Body composition may be more appropriate for confirming the association with cardiovascular diseases, including atrial fibrillation (AF). This study aimed to examine the association between body composition and the occurrence of AF.

**Methods:**

A total of 2,673,108 participants (48.6% women) without AF at baseline from the Korean national health insurance data were included. Body composition including appendicular skeletal muscle mass, body fat mass, and lean body mass were indirectly measured through validated anthropometric prediction equations. The diagnosis of AF and comorbidities were defined.

**Results:**

With a median of 9.5 (interquartile range 9.2–10.1) years’ follow-up, 25,841 (0.96%) cases of incident AF were included. In multivariable analysis, higher appendicular skeletal muscle was related to low risk of AF [hazard ratio (HR) 0.829, 95% confidence interval (CI) 0.753–0.912 for men (fifth quintile) and HR 0.888, 95% CI 0.792–0.995 for women (fifth quintile)]. In contrast, a higher body fat mass [HR 1.345, 95% CI 1.221–1.483 for men (fifth quintile) and HR 1.420, 95% CI 1.274–1.591 for women (fifth quintile)] and lean body mass [HR 2.241, 95% CI 2.182–2.303 for men (fifth quintile) and HR 1.516, 95% CI 1.368–1.667 for women (fifth quintile)] were associated with the occurrence of AF.

**Conclusions:**

In this study, body composition parameters were associated with the occurrence of AF. It should be noted that when appendicular skeletal muscle mass decreases and body fat mass and lean body mass increase, the risk of AF may be increased in general population except underweighted BMI group.

## 1. Introduction

Atrial fibrillation (AF) is the most common cardiac arrhythmia that increases the possibility of mortality, stroke, and systemic embolism (1). Globally, the present age has entered the age of aging, and the incidence of vascular risk factors has also increased. These changes have caused the incidence of AF to increase in Asian and Western populations continuously (2, 3). Therefore, it is important to control and recognize the association or risk factors for the occurrence of AF. To date, risk factors for AF, including hypertension, diabetes mellitus, coronary artery occlusive disease, aortic atheroma, poor oral hygiene, smoking, and cardiomyopathy, have been suggested. However, information regarding further modifiable associative or risk factors for AF is still lacking (4–6).

Obesity is explained as an excess of health-impairing fat mass and is commonly defined as a body mass index (BMI) ≥30 kg/m^2^ (7). The worldwide prevalence of obesity has increased over the past few decades, and the global burden of obesity is still increasing (8). It is well known that obesity, especially high levels of fat mass, worsens cardiovascular risk factors, including hypertension, lipoprotein metabolism, insulin resistance, and inflammation (9, 10). Considering the association of obesity with AF, obesity is associated with an increase in AF (11, 12). Therefore, it is important to develop an accurate method that measures obesity. Although BMI is frequently used to define obesity, the results of studies using BMI to determine the relationship between obesity and AF are inconsistent. Previous studies that defined obesity as an increased BMI showed a positive correlation between obesity and the risk of AF (13). On the other hand, other studies have showed the obesity paradox, where obese and overweight patients with AF, have a better prognosis than their leaner counterparts (14, 15). Therefore, body composition parameters, not simply BMI, may be more appropriate to confirm the association with disease (16).

To date, studies regarding the relationship between body composition and AF have been limited. In a previous study, increased body fat indices, independent of BMI, were related to the incidence of AF (17). Moreover, another study showed that high lean body mass was a determinant of AF incidence in postmenopausal women (18). Nevertheless, there have been few studies of longitudinal design targeting a general population of large sample sizes on the relationship of body composition with the occurrence of AF. Therefore, we aimed to investigate the association of predicted appendicular skeletal muscle mass index (pASMMI), predicted body fat mass index (pBFMI), and predicted lean body mass index (pLBMI), which were derived from an equation previously validated in the Korean population (16), with the occurrence of AF in a longitudinal setting.

## 2. Methods

### 2.1. Participants

**Figure 1:**
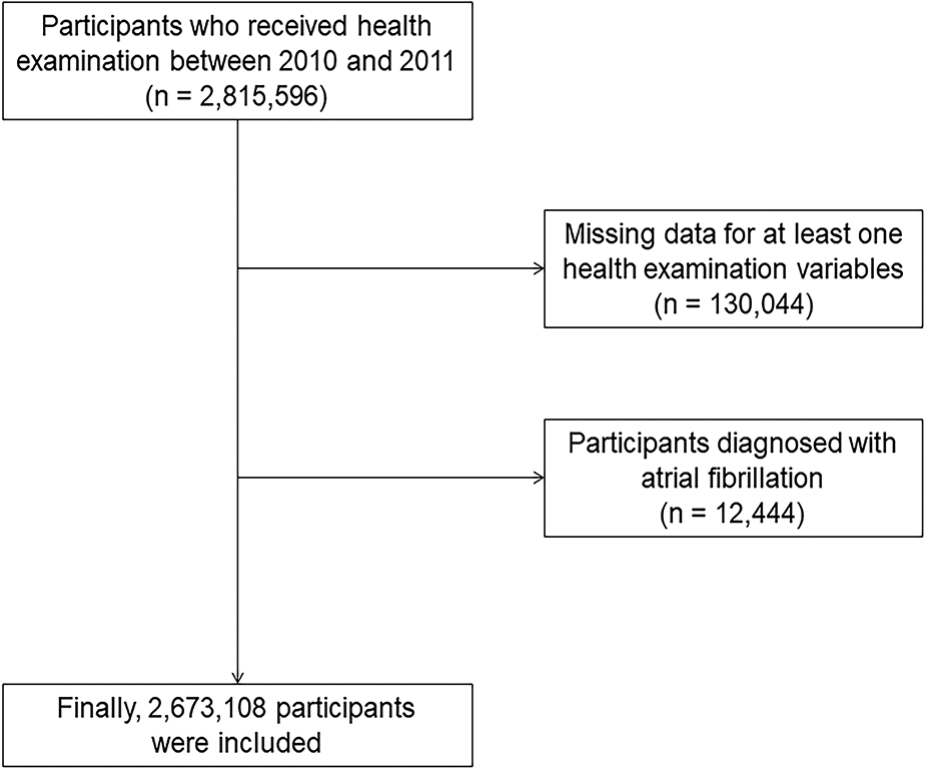
Flowchart of participant selection.

This study was performed using the National Health Insurance Service Health Screening dataset (NHIS-HEALS) provided by the Korean government. In South Korea, adults over the age of 20 are supported to undergo free health screening every other year. The Korean government combined the results of national health screening with age, sex, and sociodemographic data as well as the national health claim data, which 97% of the South Korean population are enrolled, including household income and medical history, which includes diagnostic codes, medication prescriptions, treatment or procedure information, hospitalization, and date of mortality (19). Of these data, 2,815,596 participants who underwent a national health examination between 2010 and 2011 and aged between 20 and 79 years were used to construct a dataset (NHIS-2021-1-715) and used it through a predetermined identification and validation process (6, 20). Among 2,815,596 participants, those (*n* = 130,044) with at least one missing value regarding demographic data, lifestyle, and laboratory findings were excluded. Patients with a previous history of AF (*n* = 12,444) were excluded. Finally, 2,673,108 participants were included in this study (Figure 1). Our study was permitted by the Institutional Review Board of Ewha Womans University Seoul Hospital (Institutional Review Board approval number: SEUMC 2022-02-018).

### 2.2. Predicted body composition and covariates

pASMMI, pBFMI, and pLBMI were investigated using validated anthropometric prediction equations from the Korean National Health and Nutrition Examination Survey cohort (16). In a previous study, body fat mass, lean body mass, and appendicular skeletal muscle mass were identified using dual-energy x-ray absorptiometry, and a prediction equation was constructed using different combinations of age, height, weight, level of serum creatinine, waist circumference, and lifestyle factors (physical activity, alcohol use, and smoking habit) as predictor variables (16). The combination of age, height, weight, waist circumference, serum creatinine level, and lifestyle factors were chosen in this study to create equations for the body composition. This predictive model was validated as having high predictive power, a moderate agreement rate, and low bias in the Korean general population. Appendicular skeletal muscle mass, lean body mass, and body fat mass were presented as an index (weight [kg] divided by height squared [m^2^]) for pASMMI, pBFMI, and pLBMI, respectively (Supplementary Methods 1). Predicted body composition including pASMMI, pBFMI, and pLBMI divided into quintiles (Supplementary Methods 2).

Detailed definitions of covariates are provided in Supplementary Methods 3 and previous studies (21). Variables including age, sex, BMI, household income (quartiles), smoking status (never, former, or current), alcohol consumption (none, moderate, or heavy), physical activity (low or moderate), comorbidities (hypertension, diabetes mellitus, dyslipidemia, cancer, renal disease, chronic obstructive pulmonary disease, obstructive sleep apnea syndrome, peripheral arterial disease, heart failure, and liver disease) were collected (6, 22, 23). Comorbidities were defined considering the International Classification of Diseases, Tenth Revision (ICD-10) codes, medication history, and laboratory findings from health examinations (6, 22).

### 2.3. Outcomes

The primary outcome was an occurrence. The index date was considered the date of the national health examination. If more than one check-up was performed between 2010 and 2011, the most recent health examination results were used for statistical analysis. The diagnostic accuracy of the ICD-10 code (I48) for AF in the NHIS has been validated (94%) (6). Follow-up was carried out until 31 December 2020 or until the first occurrence of death or AF.

### 2.4. Statistical analysis

Chi-squared tests and independent t-tests were performed to compare and categorical and continuous variables, respectively. For categorical variables, we tested the proportional hazard assumption using the Schoenfeld residuals. No departure from the proportional hazard assumption was detected (Supplementary Methods 4). The Cox proportional hazards model presented with hazard ratios (HR) with 95% confidence intervals (CI) was used to estimate the effect of body composition, pASMMI, pBFMI, and pLBMI, on the incidence of AF. In multivariable analysis, the following potential confounders were adjusted: BMI, household income, hypertension, diabetes mellitus, dyslipidemia, cancer, renal disease, chronic obstructive pulmonary disease, obstructive sleep apnea syndrome, peripheral arterial disease, heart failure, and liver disease. Generally standardized criteria of muscle and fat masses are not available currently. Sarcopenia is defined by authorized working groups such as the European Working Group on Sarcopenia in Older Persons and the Asian Working Group for Sarcopenia as the lowest quintile of study population (24, 25). According to these previous studies, we defined the lowest (first) quintile of pASMMI, pBFMI, and pLBMI from the study population as the reference group. For the sensitivity analysis, regression methods of Fine and Gray for competing risk data (death was a competing event for AF) were utilized according to sex. Subgroup analyses were performed with pASMMI, pBFMI, and pLBMI on the incidence of AF according to BMI categories; underweight (<18.5 kg/m^2^), normal weight (18.5–24.99 kg/m^2^), overweight (25–29.99 kg/m^2^), and obesity (≥30 kg/m^2^). Statistical analyses were performed using the SAS 9.4 version (SAS Inc., Cary, NC, USA) and R software, version 3.3.3 (R Foundation for Statistical Computing, Vienna, Austria). Two-sided *P*-values less than 0.05 were considered significant.

## 3. Results

Table 1 shows the results of comparing the baseline characteristics of 2,673,108 participants according to sex (*n* = 1,375,179 for men, *n* = 1,297,929 for women). The mean age of men was 47.47 ± 13.74 years and of women was 49.76 ± 14.28 years. Significant differences in BMI, smoking status, alcohol consumption, household income, physical activity, and comorbidities were observed between men and women (Table 1). According to BMI categories, the proportion of underweight, normal, overweight and obesity were as follows 3.7% (2.1% for men and 5.3% for women), 63.2% (59.3% for men and 67.3% for women), 29.3% (34.6% for men and 23.8% for women), and 3.8% (4.0% for men and 3.6% for women), respectively. The pASMMI (8.28 ± 0.78 kg/m^2^ for men vs. 6.26 ± 0.57 kg/m^2^ for women), pBFMI (5.39 ± 1.43 kg/m^2^ for men vs. 7.64 ± 1.94 kg/m^2^ for women), and pLBMI (18.64 ± 1.68 kg/m^2^ for men vs. 15.40 ± 1.39 kg/m^2^ for women) were significantly different according to sex (*P* < 0.001).

**Table 1:** Baseline characteristics of the study participants.

Variable	Total	Men	Women	*P*-value
Number of participants	2,673,108	1,375,179	1,297,929	
Age, years	48.58 ± 14.05	47.47 ± 13.74	49.76 ± 14.28	<.001
Body mass index (kg/m^2^)	23.74 ± 3.27	24.24 ± 3.09	23.22 ± 3.36	<.001
Household income				<.001
First quartile, lowest	502,975 (18.82)	203,592 (14.80)	299,383 (23.07)	
Second quartile	575,250 (21.52)	281,507 (20.47)	293,743 (22.63)	
Third quartile	722,093 (27.01)	399,920 (29.08)	322,173 (24.82)	
Fourth quartile, highest	872,790 (32.65)	490,160 (35.64)	382,630 (29.48)	
Smoking status				<.001
Never	1,646,321 (61.59)	418,419 (30.43)	1,227,902 (94.60)	
Former	392,042 (14.67)	368,402 (26.79)	23,640 (1.82)	
Current	634,745 (23.75)	588,358 (42.78)	46,387 (3.57)	
Alcohol consumption (drinks/week)				<.001
None	1,426,626 (53.37)	453,292 (32.96)	973,334 (74.99)	
Moderate	711,582 (26.62)	490,812 (35.69)	220,770 (17.01)	
Heavy	534,900 (20.01)	431,075 (31.35)	103,825 (8.00)	
Physical activity (min/week)				<.001
Low	1,606,512 (60.10)	779,725 (56.70)	826,787 (63.70)	
Moderate	1,066,596 (39.90)	595,454 (43.30)	471,142 (36.30)	
Comorbidities				
Hypertension	723,813 (27.08)	380,805 (27.69)	343,008 (26.43)	<.001
Diabetes mellitus	356,894 (13.35)	199,744 (14.52)	157,150 (12.11)	<.001
Dyslipidemia	739,038 (27.65)	355,543 (25.85)	383,495 (29.55)	<.001
Cancer	68,694 (2.57)	31,074 (2.26)	37,620 (2.90)	<.001
Renal disease	240,220 (8.99)	107,009 (7.78)	133,211 (10.26)	<.001
COPD	45,053 (1.69)	27,048 (1.97)	18,005 (1.39)	<.001
OSAS	909 (0.03)	763 (0.06)	146 (0.01)	<.001
Peripheral arterial disease	58,460 (2.19)	26,592 (1.93)	31,868 (2.46)	<.001
Heart Failure	27,679 (1.04)	12,049 (0.88)	15,630 (1.20)	<.001
Liver disease	405,972 (15.19)	216,630 (15.75)	189,342 (14.59)	<.001
Predicted body composition index				
pASMMI (kg/m^2^)	7.30 ± 1.22	8.28 ± 0.78	6.26 ± 0.57	<.001
pBFMI (kg/m^2^)	6.49 ± 2.04	5.39 ± 1.43	7.64 ± 1.94	<.001
pLBMI (kg/m^2^)	17.07 ± 2.24	18.64 ± 1.68	15.40 ± 1.39	<.001

*P*-values are obtained using Student's *t*-test and Chi-square test. Data are expressed as the mean ± standard deviation or *n* (%). COPD, chronic obstructive pulmonary disease; OSAS, obstructive sleep apnea syndrome; pASMMI, predicted appendicular skeletal muscle mass index; pBFMI, predicted body fat mass index; pLBMI, predicted lean body mass index.

In multivariable analysis, higher pASMMI was related to low risk of AF regardless of sex [HR 0.829, 95% CI 0.753–0.912, *P* = 0.001 for men (fifth quintile) and HR 0.888, 95% CI 0.792–0.995, *P* = 0.041 for women (fifth quintile)] (Table 2). In contrast, higher pBFMI [HR 1.345, 95% CI 1.221–1.483, *P* < 0.001 for men (fifth quintile) and HR 1.420, 95% CI 1.274–1.591, *P* < 0.001 for women (fifth quintile)] and pLBMI [HR 2.241, 95% CI 2.182–2.303, *P* < 0.001 for men (fifth quintile) and HR 1.516, 95% CI 1.368–1.667, *P* < 0.001 for women (fifth quintile)] were positively related to an increased possibility of AF regardless of sex (Table 2). Similar associations were also observed after considering the competing risk of mortality (Table 3).

**Table 2:** Hazard ratios with 95% confidence interval for atrial fibrillation in multivariable Cox proportional hazards model.

Variable	Men		Variable	Women	
Predicted body composition index (kg/m^2^)	Adjusted HR (95% CI)	*P*-value	Predicted body composition index (kg/m^2^)	Adjusted HR (95% CI)	*P*-value
pASMMI			pASMMI		
First quintile (3.97–7.64)	1 (Reference)		First quintile (3.56–5.78)	1 (Reference)	
Second quintile (7.64–8.05)	0.939 (0.895–0.985)	0.009	Second quintile (5.78–6.06)	0.906 (0.846–0.971)	0.005
Third quintile (8.05–8.42)	0.914 (0.863–0.968)	0.002	Third quintile (6.06–6.33)	0.904 (0.840–0.973)	0.007
Fourth quintile (8.42–8.71)	0.878 (0.819–0.941)	0.003	Fourth quintile (6.33–6.69)	0.928 (0.854–0.098)	0.003
Fifth quintile (8.71–21.86)	0.829 (0.753–0.912)	0.001	Fifth quintile (6.69–14.85)	0.888 (0.792–0.995)	0.041
pBFMI			pBFMI		
First quintile (≥4.21)	1 (Reference)		First quintile (≥5.99)	1 (Reference)	
Second quintile (4.21–5.00)	1.035 (0.979–1.093)	0.223	Second quintile (5.99–6.98)	1.151 (1.076–1.231)	<.001
Third quintile (5.00–5.68)	1.086 (1.020–1.156)	0.009	Third quintile (6.98–7.93)	1.253 (1.131–1.397)	<.001
Fourth quintile (5.68–6.50)	1.187 (1.103–1.266)	<.001	Fourth quintile (7.93–9.15)	1.384 (1.267–1.503)	<.001
Fifth quintile (6.50≤)	1.345 (1.221–1.483)	<.001	Fifth quintile (9.15≤)	1.420 (1.274–1.591)	<.001
pLBMI			pLBMI		
First quintile (9.07–17.26)	1 (Reference)		First quintile (9.48–14.22)	1 (Reference)	
Second quintile (17.26–18.16)	1.247 (1.124–1.371)	<.001	Second quintile (14.22–14.93)	1.156 (1.081–1.227)	<.001
Third quintile (18.16–18.95)	1.452 (1.381–1.521)	<.001	Third quintile (14.93–15.60)	1.253 (1.167–1.305)	<.001
Fourth quintile (18.95–19.93)	1.697 (1.277–2.106)	<.001	Fourth quintile (15.60–16.47)	1.335 (1.234–1.433)	<.001
Fifth quintile (19.93–48.50)	2.241 (2.182–2.303)	<.001	Fifth quintile (16.47–35.91)	1.516 (1.368–1.667)	<.001

A multivariable model is used to determine the association of predicted body composition index with the development of atrial fibrillation adjusted for body mass index, household income, hypertension, diabetes mellitus, dyslipidemia, cancer, renal disease, chronic obstructive pulmonary disease, obstructive sleep apnea syndrome, peripheral arterial disease, heart failure, and liver disease. HR, hazard ratio; CI, confidence interval; pASMMI, predicted appendicular skeletal muscle mass index; pBFMI, predicted body fat mass index; pLBMI, predicted lean body mass index.

**Table 3:** Hazard ratios with 95% confidence interval for atrial fibrillation in competing risk analysis with fine - gray model.

Variable	Men		Variable	Women	
Predicted body composition index (kg/m^2^)	Adjusted HR (95% CI)	*P*-value	Predicted body composition index (kg/m^2^)	Adjusted HR (95% CI)	*P*-value
pASMMI			pASMMI		
First quintile (3.97–7.64)	1 (Reference)		First quintile (3.56–5.78)	1 (Reference)	
Second quintile (7.64–8.05)	0.968 (0.924–1.014)	0.172	Second quintile (5.78–6.06)	0.973 (0.919–1.032)	0.289
Third quintile (8.05–8.42)	0.936 (0.886–0.989)	0.019	Third quintile (6.06–6.33)	0.930 (0.902–0.962)	0.007
Fourth quintile (8.42–8.71)	0.884 (0.827–0.944)	0.003	Fourth quintile (6.33–6.69)	0.889 (0.831–0.943)	0.006
Fifth quintile (8.71–21.86)	0.801 (0.731–0.877)	<.001	Fifth quintile (6.69–14.85)	0.894 (0.842–0.946)	0.003
pBFMI			pBFMI		
First quintile (≥4.21)	1 (Reference)		First quintile (≥5.99)	1 (Reference)	
Second quintile (4.21–5.00)	1.067 (1.009–1.127)	0.022	Second quintile (5.99–6.98)	1.019 (0.918–1.123)	0.624
Third quintile (5.00–5.68)	1.125 (1.056–1.199)	0.003	Third quintile (6.98–7.93)	1.049 (0.969–1.136)	0.237
Fourth quintile (5.68–6.50)	1.226 (1.138–1.320)	<.001	Fourth quintile (7.93–9.15)	1.106 (1.012–1.208)	0.026
Fifth quintile (6.50≤)	1.362 (1.234–1.503)	<.001	Fifth quintile (9.15 ≤)	1.114 (1.001–1.223)	0.021
pLBMI			pLBMI		
First quintile (9.07–17.26)	1 (Reference)		First quintile (9.48–14.22)	1 (Reference)	
Second quintile (17.26–18.16)	1.206 (1.082–1.323)	<.001	Second quintile (14.22–14.93)	1.122 (1.097–1.149)	<.001
Third quintile (18.16–18.95)	1.322 (1.252–1.393)	<.001	Third quintile (14.93–15.60)	1.241 (1.193–1.293)	<.001
Fourth quintile (18.95–19.93)	1.594 (1.174–2.013)	<.001	Fourth quintile (15.60–16.47)	1.258 (1.234–1.284)	<.001
Fifth quintile (19.93–48.50)	2.121 (1.723–2.523)	<.001	Fifth quintile (16.47–35.91)	1.306 (1.288–1.327)	<.001

A multivariable model is used to determine the association of predicted body composition index with the development of atrial fibrillation adjusted for body mass index, household income, hypertension, diabetes mellitus, dyslipidemia, cancer, renal disease, chronic obstructive pulmonary disease, obstructive sleep apnea syndrome, peripheral arterial disease, heart failure, and liver disease. HR, hazard ratio; CI, confidence interval; pASMMI, predicted appendicular skeletal muscle mass index; pBFMI, predicted body fat mass index; pLBMI, predicted lean body mass index.

**Figure 2:**
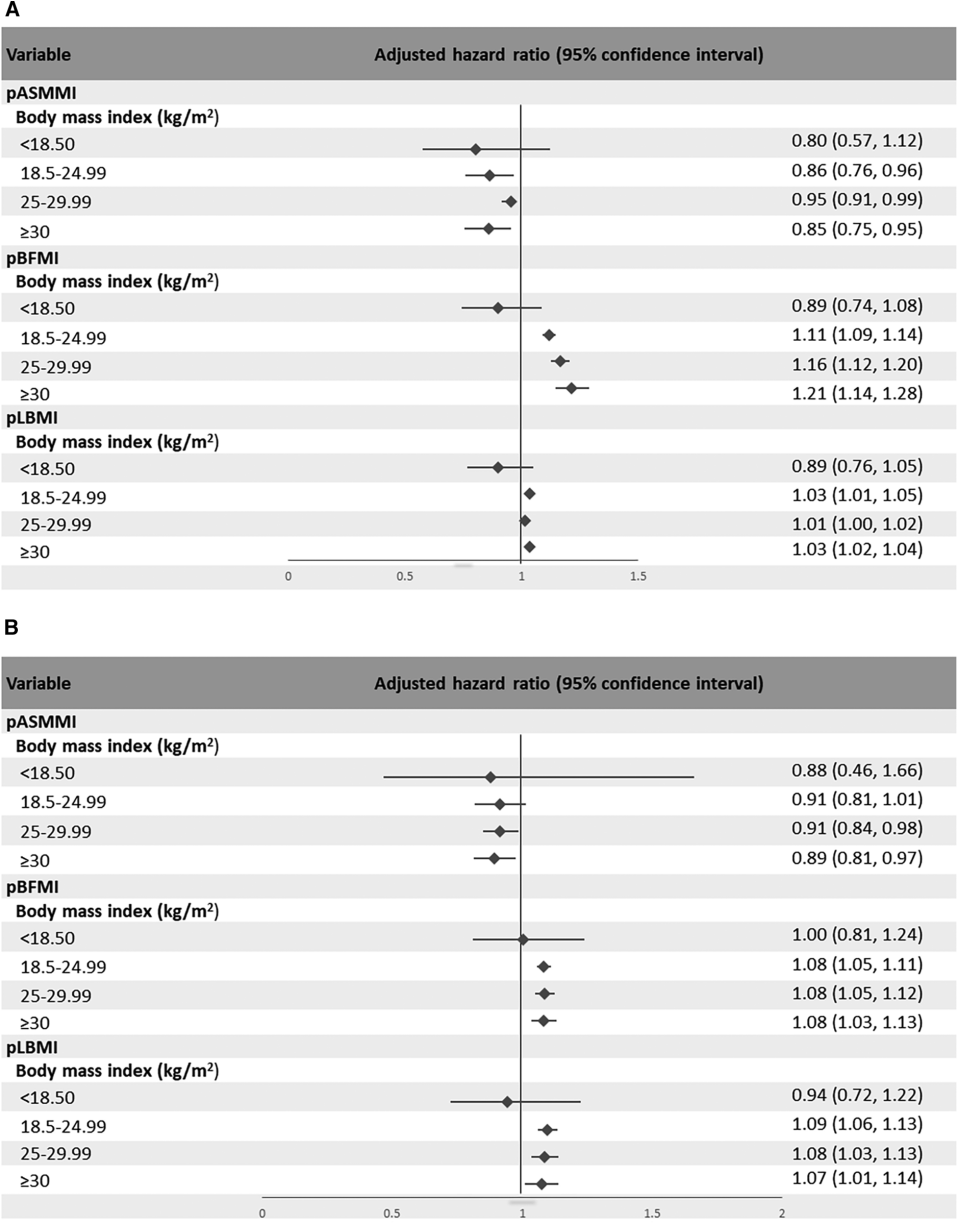
Forest plot showing the hazard ratio and 95% confidence intervals of the association between body composition indices according to subcategories of body mass index and atrial fibrillation for (**A**) men and (**B**) women.

The results for the association of body composition with incident AF according to BMI subgroups are shown in Table 4 and Figures 2A,B. A higher pASMMI was related to a decreased possibility of AF in both sexes. In contrast, a higher pLBMI was also related to an increased risk of AF, except the underweight group in both sexes. Moreover, increased pBFMI was related to the incidence of AF, except the underweight group in men.

**Table 4:** Subgroup analysis according to body mass index categories regarding predicted body composition indices and atrial fibrillation.

Variable	Men		Women	
	HR (95% CI)	*P*-value	HR (95% CI)	*P*-value
Body mass index (kg/m^2^)				
<18.50				
pASMMI	0.804 (0.577–1.121)	0.198	0.880 (0.465–1.662)	0.692
pBFMI	0.899 (0.743–1.088)	0.273	1.002 (0.811–1.239)	0.982
pLBMI	0.897 (0.767–1.050)	0.176	0.944 (0.726–1.226)	0.663
18.50–24.99				
pASMMI	0.863 (0.760–0.965)	0.014	0.915 (0.816–1.015)	0.095
pBFMI	1.118 (1.092–1.145)	<.001	1.084 (1.058–1.111)	<.001
pLBMI	1.034 (1.013–1.056)	0.002	1.097 (1.060–1.135)	<.001
25–29.99				
pASMMI	0.955 (0.914–0.993)	0.030	0.915 (0.849–0.986)	0.019
pBFMI	1.166 (1.128–1.205)	<.001	1.087 (1.050–1.126)	<.001
pLBMI	1.015 (1.005–1.025)	0.018	1.085 (1.034–1.138)	0.001
≥30				
pASMMI	0.857 (0.756–0.954)	0.034	0.894 (0.814–0.976)	<.001
pBFMI	1.215 (1.145–1.289)	<.001	1.083 (1.036–1.132)	0.004
pLBMI	1.034 (1.022–1.046)	0.025	1.075 (1.012–1.141)	0.019

A multivariable model is used to determine the association of predicted body composition index with the development of atrial fibrillation adjusted for body mass index, household income, hypertension, diabetes mellitus, dyslipidemia, cancer, renal disease, chronic obstructive pulmonary disease, obstructive sleep apnea syndrome, peripheral arterial disease, heart failure, and liver disease. HR, hazard ratio; CI, confidence interval; pASMMI, predicted appendicular skeletal muscle mass index; pBFMI, predicted body fat mass index; pLBMI, predicted lean body mass index.

## 4. Discussion

The main finding of our study was that pASMMI was negatively associated with the risk of AF, and pBFMI and pLBMI were positively associated with the incidence of AF.

Generally, skeletal muscle mass is considered beneficial to health. However, it is rarely known how skeletal muscle mass affects for risk of AF. A previous study showed that patients with AF have a lower percentage of skeletal muscle mass (26). In addition, both sarcopenia and the ratio of muscle components are associated with electrocardiogram abnormalities, including AF (27). In line with previous report that sarcopenic overweight/obese was associated with risk of AF (28) and sarcopenia was associated with cardiovascular disease including AF in older patients (27), our report that pASMMI showed a negative association with risk of AF regardless BMI. Because skeletal muscle generates considerable metabolic and oxygen demand in the body, it affects cardiac muscle, cardiac output, and heart rate (29).

Many previous epidemiologic studies have shown that obesity increases the risk of AF; however, confusion, including unexpected J- or U-shaped associations, has often been observed (30). Some studies in relation to obesity showed that greater lean body mass was a strong independent risk factor for AF, and fat mass was also related to a higher risk of AF (17, 18, 31, 32). Although previous studies showed that body fat composition/distribution, which may be linked to non-alcoholic fatty liver disease (NAFLD), has been associated with a higher occurrence of AF (33, 34), our study showed that body fat composition was associated with occurrence of AF after adjusting liver disease including NAFLD. Furthermore, though several controversies exist, regardless of BMI, our study showed a positive relationship between higher pBFMI and the incidence of AF, in line with the findings of previous studies (27, 32). Body fat mass is related to an increased risk of hypertension, insulin resistance, diabetes, coronary heart disease, and heart failure, which contribute to AF (34). Furthermore, increased left atrial size, volume overload, left ventricular diastolic dysfunction, and left atrial dysfunction due to body fat mass further contribute to electrophysiological remodeling and conduction abnormalities resulting in AF (35, 36).

Furthermore, our study showed a positive relationship between pLBMI and the risk of AF, in line with the findings of previous studies (31, 32, 37). But, because participants with low lean body mass were a relatively unhealthy group (38), effect lowering the risk of AF might be offset in participants with underweighted BMI. Also, in present study, the absolute number of underweighted BMI groups is small. For those factors, the significant association between high lean body mass and AF risk might not be observed in underweighted BMI group. In addition, because most overweight and obese people have high predicted lean body mass and fat mass, a higher pLBMI was related to an increased risk of AF in overweight and obese participants (38).

In participants with underweight BMI, we observed a negative relationship between pLBMI and pASMMI and the risk of AF. A previous study suggested that being underweight was significantly related to an increased risk of AF (39). There are several potential mechanisms underlying this obesity paradox. An animal study suggested that the loss of myostatin, a well-known negative regulator of skeletal muscle growth that causes sarcopenia, can lead to AF (40). Studies have linked AF to a deficiency of trace elements (41). However, all of the predicted body composition indices were not significantly related to the occurrence of AF in underweight participants due to the small number of underweight participants.

The strength of our study is that it showed an association between body composition indices and AF in a large sample of the general Korean population in a longitudinal setting. Furthermore, our study could potentially serve as evidence for future randomized clinical trials investigating whether the risk of AF changes with the regulation of body composition and also facilitate future comparative research with direct measurements of body composition using dual energy x-ray absorptiometry or computed tomography scan. In addition, our findings could be practically utilized as evidence for the importance of maintaining a healthy body composition. In other words, our results could be used as educational materials for the general population, highlighting that the potential benefits of maintaining or increasing skeletal muscle mass and decreasing body fat mass cause reducing the risk of AF. However, our study has some limitations. First, although it was conducted with a longitudinal design, this is a retrospective study; therefore, we could not confirm the causal relationship or exclude confounders. Second, we used the equation for predicted body composition validated in the Korean population for large-scale analysis rather than directly conducting dual-energy x-ray absorptiometry. In addition, because the equation was estimated for the Korean population, these findings may not be generalizable to other ethnicities. Third, the predicted body composition indices were measured once or twice during the study period; thus, possible serial changes in body composition were not considered. Finally, this is an epidemiological study that cannot explain the basic mechanism of the association between body composition and AF.

In conclusion, our results demonstrated that a lower muscle mass, higher lean body mass, and higher fat mass were related to an increased risk of AF in general population except underweighted BMI group. These associations between AF and body composition differed according to the BMI categories. For studies with longitudinal settings and large sample sizes, body composition, including body fat mass, lean body mass, and skeletal muscle mass, may be more accurate in confirming the association with AF than BMI.

## Data Availability

The raw data supporting the conclusions of this article will be made available by the authors, without undue reservation.
