# Supplementary material for: Association of predicted body composition with occurrence of atrial fibrillation
Source: Front Cardiovasc Med. 2023 Oct 10;10:1159087. doi: 10.3389/fcvm.2023.1159087 (PMC10595030; doi:10.3389/fcvm.2023.1159087)
Supplement: Supplementary file 1 [file Datasheet1.pdf]

Supplementary Methods 1. Equations for predicted appendicular skeletal muscle mass index, predicted body fat mass index, and predicted lean body mass index

predicted appendicular skeletal muscle mass index

<men> – 2.236 – 0.011×(age) + 0.081×(height, cm) + 0.324×(weight, kg) – 0.121×(waist circumference, cm) – 0.008×(level of serum creatinine, mg/dL) + 0.200×(moderate physical activity) + 0.587×(vigorous physical activity) – 0.195×(past smoker) – 0.016×(current smoker) + 0.004×(moderate drinker) + 0.151×(heavy drinker)

<women> – 8.447 + 0.002×(age) + 0.091×(height, cm) + 0.203×(weight, kg) – 0.034×(waist circumference, cm) + 0.539×(level of serum creatinine, mg/dL) + 0.103×(moderate physical activity) + 0.362×(vigorous physical activity) – 0.203×(past smoker) + 0.000×(current smoker) + 0.103×(moderate drinker) + 0.031×(heavy drinker)

predicted body fat mass index

<men> 0.561 – 0.012×(age) – 0.133×(height, cm) + 0.310×(weight, kg) + 0.199×(waist circumference, cm) + 0.253×(level of serum creatinine, mg/dL) – 0.247×(moderate physical activity) – 0.878×(vigorous physical activity) + 0.708×(past smoker) – 0.275×(current smoker) + 0.077×(moderate drinker) – 0.291×(heavy drinker)

<women> 12.269 – 0.014×(age) – 0.172×(height, cm) + 0.530×(weight, kg) + 0.058×(waist circumference, cm) – 0.314×(level of serum creatinine, mg/dL) – 0.123×(moderate physical activity) – 0.541×(vigorous physical activity) + 0.261×(past smoker) – 0.242×(current smoker) – 0.218×(moderate drinker) – 0.182×(heavy drinker)

predicted lean body mass index

<men> – 0.296 + 0.012×(age) + 0.134×(height, cm) + 0.675×(weight, kg) – 0.201×(waist

circumference, cm) – 0.249×(level of serum creatinine, mg/dL) + 0.270×(moderate physical activity) + 0.924×(vigorous physical activity) – 0.559×(past smoker) + 0.234×(current smoker) – 0.046×(moderate drinker) + 0.324×(heavy drinker)

<women> – 11.941 + 0.015×(age) + 0.171×(height, cm) + 0.457×(weight, kg) – 0.060×(waist circumference, cm) + 0.428×(level of serum creatinine, mg/dL) + 0.181×(moderate physical activity) + 0.654×(vigorous physical activity) – 0.254×(past smoker) + 0.221×(current smoker) + 0.209×(moderate drinker) + 0.126×(heavy drinker)

Supplementary Methods 2. Divided predicted body composition including pASMMI, pBFMI, and pLBMI into quintiles.

In men, pASMMI quintiles were 3.97 kg/m^2 to 7.64 kg/m^2 (first quintile), 7.64 kg/m^2 to 8.05 kg/m^2 (second quintile), 8.05 kg/m^2 to 8.42 kg/m^2 (third quintile), 8.42 kg/m^2 to 8.71 kg/m^2 (fourth quintile), and 8.71 kg/m^2 to 21.86 kg/m^2 (fifth quintile). In women, pASMMI quintiles were 3.56 kg/m^2 to 5.78 kg/m^2 (first quintile), 5.78 kg/m^2 to 6.06 kg/m^2 (second quintile), 6.06 kg/m^2 to 6.33 kg/m^2 (third quintile), 6.33 kg/m^2 to 6.69 kg/m^2 (fourth quintile), and 6.69 kg/m^2 to 14.85 kg/m^2 (fifth quintile). Furthermore, pBFMI quintiles in men were 4.21 kg/m^2 or lower (first quintile), 4.21 kg/m^2 to 5.00 kg/m^2 (second quintile), 5.00 kg/m^2 to 5.68 kg/m^2 (third quintile), 5.68 kg/m^2 to 6.50 kg/m^2 (fourth quintile), and greater than 6.50 kg/m^2 (fifth quintile) and pBFMI quintiles in women were 5.99 kg/m^2 or lower (first quintile), 5.99 kg/m^2 to 6.98 kg/m^2 (second quintile), 6.98 kg/m^2 to 7.93 kg/m^2 (third quintile), 7.93 kg/m^2 to 9.15 kg/m^2 (fourth quintile), and greater than 9.15 kg/m^2 (fifth quintile). In addition, pLBMI quintiles in men were 9.07 kg/m^2 to 17.26 kg/m^2 (first quintile), 17.26 kg/m^2 to 18.16 kg/m^2 (second quintile), 18.16 kg/m^2 to 18.95 kg/m^2 (third quintile), 18.95 kg/m^2 to 19.93 kg/m^2 (fourth quintile), and 19.93 kg/m^2 to 48.50 kg/m^2 (fifth quintile) and pLBMI quintiles in women were 9.48 kg/m^2 to 14.22 kg/m^2 (first quintile), 14.22 kg/m^2 to 14.93 kg/m^2 (second quintile), 14.93 kg/m^2 to 15.60 kg/m^2 (third quintile), 15.60 kg/m^2 to 16.47 kg/m^2 (fourth quintile), and 16.47 kg/m^2 to 35.91 kg/m^2 (fifth quintile).

Supplementary Methods 3. Definition of covariates

Smoking status

Definition and measurement of variables like usual smoking status were obtained by questionnaire in the health examination program. Smoking status was used to categorize participants into three groups: none, former smoker, and current smoker. Current smoker was classified according to the WHO definition as a person who has smoked more than five packs (100 cigarettes) in a lifetime and smoked daily or occasionally for the last 28 days. Former smoker was defined as a person who had smoked more than 100 cigarettes in a lifetime and had not smoked in the last 28 days (1).

Alcohol consumption

Definition and measurement of variables like usual alcohol consumption were obtained by questionnaire in the health examination program. Alcohol consumption was categorized into three groups: none, moderate drinker, and heavy drinker. Differentiation between moderate and heavy drinker was based on whether a patient usually takes more than 14 drinks/7 drinks per week for men/women. The drinks were calculated by multiplying the average drinking frequency per week by the number of drinks per occasion.

Physical activity

Physical activity was assessed using the Korean version of the International Physical Activity Questionnaire-short form. We created composite physical activity based on Metabolic Equivalent Task (MET)-minutes/week (walking: 3.3 METs; moderate physical activity: 4.0 METs; vigorous physical activity: 8.0 METs), which was categorized as follows based on

total physical activity metabolic equivalents: low (< 600 METs), moderate (600–2,999 METs), and vigorous (≥ 3,000 METs) (2, 3).

Hypertension

Hypertension was defined as using at least one claim of International Classification of Diseases, Tenth Revision (ICD-10) code (I10-15) with the prescription of an anti-hypertensive agent, claims of ICD-10 code (I10-15) more than two times, a systolic blood pressure of ≥140 mmHg and a diastolic blood pressure of ≥90 mmHg or positive checking in self-report questionnaire on hypertension in the health examination program.

Diabetes mellitus

Diabetes mellitus was as defined using at least one claim of ICD-10 code (E11-14) with the prescription of a anti-diabetic agent, claims of ICD-10 code (E11-14) more than two times, fasting serum glucose concentration of ≥7.0 mmol/L or positive checking in self-report questionnaire on diabetes mellitus in the health examination program.

Dyslipidemia

Dyslipidemia was defined as using at least one claim of ICD-10 code (E78) with the prescription of an anti-dyslipidemic agent, claims of ICD-10 code (E78) more than two times or total cholesterol level of ≥240 mg/dL.

Cancer

Cancer was defined as using claims of ICD-10 code (C00–C97) more than two times with cancer specific deductible code (V027, V193-4) from the Health Insurance Review and Assessment Service.

Renal disease

Renal disease was defined as using claims of ICD–10 codes (N17-19, I12-13, E08.2, E10.2, E11.2, E13.2) more than two times or estimated glomerular filtration rate of <60 $mL/min/1.73m^2$.

Chronic obstructive pulmonary disease

Chronic obstructive pulmonary disease was defined as using claims of ICD–10 codes (J41, J42, J43, or J44) more than two times (4).

Obstructive sleep apnea syndrome

Obstructive sleep apnea syndrome was defined as using claims of ICD–10 codes (G47.3) more than two times (4).

Peripheral arterial disease

Peripheral arterial disease was defined as using claims of ICD–10 codes (I70, I73) more than two times (5).

Heart failure

Heart failure was defined as using claims of ICD–10 codes (I50) more than two times (5).

liver disease

Liver disease was defined as using claims of ICD–10 codes (K70-K77, B15-19, C22, C22.9, E83.0, E83.1) more than two times.

References

1. Lee KH, Lee CM, Kwon HT, Oh S-W. Relationship between Obesity and Smoking in Korean Men: Data Analyses from the Third and Fourth Korea National Health and Nutrition Examination Surveys (KNHANES). JKSRNT. 2010;1(2):115-23.

2. Do-Hyun K, Eun-Joon L, Ji-Yeon L, Duk-Chul L. The association and the characteristics of the smoking status and differences in physical activity level in Korean adults: The Sixth Korea National Health and Nutrition Examination Survey (KNHANES VI-1), 2013. KJFP. 2015;5(3):510-6.

3. Oh JY, Yang YJ, Kim BS, Kang JH. Validity and Reliability of Korean Version of International Physical Activity Questionnaire (IPAQ) Short Form. J Korean Acad Fam Med. 2007;28(7):532-41.

4. Lee EJ, Suh JD, Cho JH. The incidence of prostate cancer is increased in patients with obstructive sleep apnea: Results from the national insurance claim data 2007-2014. Medicine (Baltimore). 2021;100(6):e24659.

5. Choi EK. Cardiovascular Research Using the Korean National Health Information Database. Korean circulation journal. 2020;50(9):754-72.

Supplementary Methods 4. Test for assumption of the proportionality of hazards by Schoenfeld's residuals.

	Men			Women		
		Unadjusted model	Multivariable model		Unadjusted model	Multivariable model
	Event rate (%)	P-value	P-value	Event rate (%)	P-value	P-value
pASMMI (kg/m^2)						
First quintile	0.37%			0.33%		
Second quintile	0.32%	0.797	0.185	0.26%	0.461	0.247
Third quintile	0.30%	0.332	0.241	0.20%	0.217	0.197
Fourth quintile	0.28%	0.814	0.324	0.15%	0.307	0.106
Fifth quintile	0.25%	0.792	0.249	0.14%	0.412	0.093
pBFMI (kg/m^2)						
First quintile	0.21%			0.09%		
Second quintile	0.25%	0.841	0.451	0.14%	0.944	0.384
Third quintile	0.30%	0.204	0.276	0.19%	0.561	0.233
Fourth quintile	0.35%	0.426	0.394	0.27%	0.517	0.184
Fifth quintile	0.40%	0.107	0.089	0.37%	0.317	0.113
pLBMI (kg/m^2)						
First quintile	0.29%			0.29%		
Second quintile	0.29%	0.675	0.723	0.29%	0.317	0.246
Third quintile	0.31%	0.512	0.899	0.31%	0.457	0.208
Fourth quintile	0.32%	0.371	0.495	0.32%	0.274	0.125
Fifth quintile	0.32%	0.208	0.092	0.32%	0.086	0.079

pASMMI, predicted appendicular skeletal muscle mass index; pBFMI, predicted body fat mass index; pLBMI, predicted lean body mass index
